# Cell-Type-Specific Transcriptomic Alterations in the Epileptic Prefrontal Cortex and Complementary Neuroimaging Findings in an Independent Cohort

**DOI:** 10.7759/cureus.116642

**Published:** 2026-09-21

**Authors:** Jing Chen, Yuqi Ying, Cheng Wang, Zhoule Zhu, Jingquan Lin, Junming Zhu, Zhe Zheng

**Affiliations:** 1 Neurology, Hangzhou Hospital of Traditional Chinese Medicine, Hangzhou, CHN; 2 Urology, Renji Hospital, Shanghai Jiao Tong University School of Medicine, Shanghai, CHN; 3 Neurosurgery, The Second Affiliated Hospital, Zhejiang University School of Medicine, Hangzhou, CHN

**Keywords:** astrocytes and oligodendrocyte lineage, diffusion tensor imaging, epilepsy, prefrontal cortex, single-nucleus rna sequencing

## Abstract

Background and objectives: Epilepsy is a complex neurological disorder involving distributed neuronal and glial networks. The cellular transcriptional states associated with epilepsy and their relationship to macroscopic brain abnormalities remain incompletely characterized. We therefore performed a two-part exploratory study using a public postmortem prefrontal cortex (PFC) single-nucleus RNA sequencing (snRNA-seq) dataset and an independent clinical neuroimaging cohort.

Methods: Public snRNA-seq data from eight individuals classified as having epilepsy and seven controls were processed with CellRanger (10x Genomics, Pleasanton, California) and Seurat (Satija Lab, New York Genome Center, New York, New York). Cell-type composition, exploratory nucleus-level differential expression, Gene Ontology (GO)/Kyoto Encyclopedia of Genes and Genomes (KEGG) enrichment, Monocle pseudotime analysis, and SCENIC (Single-Cell rEgulatory Network Inference and Clustering) regulon inference were evaluated. Separately, 18F-fluorodeoxyglucose positron emission tomography-computed tomography (18F-FDG PET-CT) and diffusion tensor imaging (DTI) data from 16 patients with neocortical epilepsy were analyzed using within-patient lesion-versus-contralateral comparisons. Because the sequencing and imaging cohorts were independent, the two components were interpreted as complementary observations rather than direct cross-modal validation.

Results: After quality control, 16,132 nuclei were retained (11,587 control and 4,545 epilepsy nuclei). Oligodendrocyte-lineage representation was lower in epilepsy than in controls (5.3% vs. 14.7%; exploratory P = 0.023), whereas layer 2/3 (L2/3) excitatory neurons showed higher representation (47.7% vs. 29.5%). Exploratory cell-level enrichment analysis identified MAPK-related signaling as the top-ranked KEGG pathway among upregulated genes in L2/3 neurons, and SCENIC identified a STAT3 (signal transducer and activator of transcription 3)-associated reactive astrocytic regulon pattern. Oligodendrocyte-lineage nuclei showed altered gene-expression patterns and different distributions along an inferred pseudotime continuum; these observations were not interpreted as direct evidence of impaired differentiation. In the independent imaging cohort, PET signal was lower in epileptic lesions than in contralateral regions (P = 0.007), and fractional anisotropy (FA) was reduced (P < 0.001), whereas mean diffusivity (MD) was not significantly different (P = 0.101). PET and FA findings remained significant after Bonferroni correction for three prespecified imaging comparisons (adjusted threshold P < 0.0167).

Conclusions: The study identifies exploratory cell-type-specific transcriptional alterations in the public PFC dataset and, independently, metabolic and white matter microstructural abnormalities in a separate neocortical epilepsy cohort. These findings represent complementary observations across different biological scales rather than direct molecular-imaging validation. Larger donor-level transcriptomic studies, matched molecular-imaging cohorts, and functional experiments are required to validate the candidate cellular and molecular signatures.

## Introduction

Epilepsy is a common chronic neurological disorder characterized by an enduring predisposition to recurrent unprovoked seizures and affects approximately 65 million people worldwide [1]. Its long-term course, associated comorbidities, disability, and socioeconomic burden substantially affect patients and their families. Although antiseizure medications improve seizure control for many patients, approximately one-third do not achieve sustained seizure freedom and may meet criteria for drug-resistant epilepsy [1-3]. For selected patients with focal drug-resistant epilepsy, surgical treatment can be effective, but clinical management remains challenging because epilepsy involves complex alterations in neuronal circuits, glial cells, and distributed brain networks [4]. A better understanding of the cellular and molecular processes associated with epileptic brain remodeling is therefore important for improving disease characterization and identifying biological processes that warrant further investigation.

Seizures are also an important clinical manifestation of brain tumors. In patients with primary brain tumors, seizures may precede tumor diagnosis and have been reported as the first presenting symptom in approximately 30%-60% of cases [5]. Brain tumor-related seizures further illustrate the clinical and mechanistic heterogeneity of seizure disorders and emphasize that epileptic activity can emerge from diverse alterations in the local cellular microenvironment and broader neural networks.

Epilepsy has traditionally been viewed primarily as a disorder of abnormal neuronal excitability and synchronization. However, increasing evidence indicates that non-neuronal populations also contribute to epileptogenesis and seizure expression. Astrocytes can influence extracellular ion homeostasis, neurotransmitter recycling, energy metabolism, and neuroinflammatory signaling, while neuron-glia interactions can shape network excitability [1,6]. Oligodendrocyte-lineage and myelin abnormalities have also been reported in epileptic brain tissue, including focal cortical dysplasia, suggesting that white matter and axonal support systems may participate in network dysfunction [7]. These observations support simultaneous investigation of neuronal and glial populations rather than an exclusively neuron-centered model.

Transcriptomic studies of human epilepsy tissue have provided important information about disease-associated molecular alterations, but many previous studies relied on bulk surgical or postmortem tissue, in which expression profiles represent averages across heterogeneous cell populations [8,9]. Consequently, changes restricted to specific cell types can be obscured. Single-nucleus RNA sequencing (snRNA-seq) enables cell-type-resolved transcriptional profiling and is particularly suitable for archived or frozen human brain tissue [10,11]. Its application to neurological disease has revealed cell-specific molecular states that are not evident in bulk tissue [12]. In epilepsy, single-cell and single-nucleus approaches have demonstrated the value of resolving disease-associated neuronal subtypes and transcriptional programs [13].

The prefrontal cortex (PFC) was analyzed in the present study because the public snRNA-seq dataset used here was generated from this cortical region. Importantly, the available metadata do not establish the PFC as the seizure-onset zone in these individuals; therefore, the samples are not interpreted as confirmed epileptogenic foci. Nevertheless, frontal regions participate in distributed seizure networks, and frontal lobe epilepsy can be conceptualized as a network disorder extending from genes and local circuits to large-scale brain systems [14]. More broadly, focal epilepsy is associated with connectivity disturbances extending beyond the epileptogenic zone and with neurocognitive consequences of network reorganization [15]. Thus, cell-type-specific transcriptional alterations in the PFC may provide information about epilepsy-associated cortical network remodeling without implying that this region was the seizure-onset zone.

Neuroimaging offers a complementary view at the tissue and systems levels. Interictal 18F-fluorodeoxyglucose positron emission tomography-computed tomography (18F-FDG PET) commonly demonstrates regional hypometabolism in focal epilepsy and is used in presurgical evaluation, whereas diffusion tensor imaging (DTI) provides quantitative measures of white matter microstructural organization [16,17]. These modalities therefore provide macroscopic phenotypes that can be considered alongside molecular observations. However, direct molecular-imaging validation requires measurements from the same individuals or appropriately matched cohorts. In the present study, the neuroimaging and snRNA-seq datasets were obtained from independent populations and were therefore analyzed as separate, complementary data streams.

In this study, we designed a two-part exploratory investigation. First, we analyzed publicly available postmortem human PFC snRNA-seq data to characterize epilepsy-associated differences in cellular composition and transcriptional states, with particular attention to excitatory neurons, astrocytes, and oligodendrocyte-lineage cells. Second, we independently analyzed PET-CT and DTI data from a separate clinical cohort of patients with neocortical epilepsy to characterize regional metabolic and white matter microstructural abnormalities. Our objective was to describe potentially convergent biological themes across cellular and tissue levels while avoiding direct patient-level, causal, or molecular-imaging inference.

## Materials and methods

Public snRNA-seq dataset and clinical imaging cohort

The snRNA-seq raw data were obtained from the Sequence Read Archive (SRA; accession PRJNA434002) and originated from postmortem PFC tissue in the dataset reported by Velmeshev et al. [18]. For the present analysis, eight samples classified as epilepsy and seven control samples were included. All transcriptomic specimens analyzed here were derived from postmortem brain tissue. Detailed clinical information regarding epilepsy classification (including focal versus generalized epilepsy), seizure frequency, etiology, disease duration, the relationship of the sampled PFC region to the seizure-onset zone, and antiseizure medication exposure was not available in the public dataset. Postmortem interval (PMI) information was available and is summarized in Table 1. FASTQ reads were aligned to the human GRCh38 reference genome supplied by 10× Genomics, filtered, and counted using CellRanger version 4.0.0 (10x Genomics, Pleasanton, California) with the standard “cellranger count” workflow to generate gene-barcode matrices.

The clinical imaging data were extracted from the electronic medical record system of the Second Affiliated Hospital, Zhejiang University School of Medicine. The imaging cohort comprised 16 patients diagnosed with neocortical epilepsy who underwent PET-CT and DTI as part of their clinical evaluation. The study was approved by the Institutional Review Board of the Second Affiliated Hospital, Zhejiang University School of Medicine (2021-0110), and informed consent was obtained from the participants or their legal guardians, as appropriate. The transcriptomic and imaging cohorts were independent and had no patient-level correspondence.

Single-nucleus sequencing analysis using Seurat

The gene-barcode matrices were analyzed using Seurat version 3.2.2 (Satija Lab, New York Genome Center, New York, New York) [19]. Sample integration was performed using “FindIntegrationAnchors” and “IntegrateData” to reduce technical variation across samples. Quality-control exclusion criteria included (1) <200 or >3,000 detected genes per nucleus; (2) mitochondrial transcript content >15%; (3) ribosomal transcript content >10%; and (4) erythrocytic transcript content >5%. Genes detected in fewer than one nucleus were excluded during Seurat object construction. After quality control, 16,132 nuclei were retained, including 11,587 from control individuals and 4,545 from individuals classified as having epilepsy.

Data were log-normalized using “NormalizeData,” highly variable genes were identified using “FindVariableFeatures,” and principal component analysis was performed with “RunPCA.” The top 30 principal components were used for neighbor graph construction and clustering with “FindNeighbors” and “FindClusters” at a resolution of 0.5. Excitatory neurons, astrocytes, and oligodendrocyte-lineage cells were subsequently subsetted and re-clustered at resolutions of 0.2, 0.5, and 0.2, respectively. Clusters were visualized with t-distributed stochastic neighbor embedding (t-SNE). Cell identities were assigned using cluster-associated genes together with canonical central nervous system markers. Representative markers included RBFOX3/CUX2/RORB/TLE4/NRGN for excitatory neuronal subclasses, SLC1A2/GFAP-associated patterns for astrocytic populations, and PLP1/MBP/PDGFRA/OLIG2 for oligodendrocyte-lineage populations. Expression patterns were visualized using “VlnPlot,” “DotPlot,” “DoHeatmap,” and “FeaturePlot.”

Because the numbers of retained nuclei differed substantially between disease groups, all nuclei meeting the predefined quality-control criteria were retained, and no group-level downsampling or bootstrap resampling was performed. Seurat integration was used to reduce technical variation but was not considered a correction for unequal group-level nuclei numbers. Accordingly, analyses of cluster representation, rare cellular states, and nucleus-level differential expression were treated as exploratory.

Pseudotime trajectory analysis

Pseudotime analysis was performed using Monocle version 2.16.0 (Trapnell Lab, University of Washington, Seattle, Washington) [20]. Oligodendrocyte precursor cell (OPC) and oligodendrocyte nuclei were isolated from the integrated object. Highly dispersed genes were used as ordering features, and trajectories were constructed using “setOrderingFilter,” “reduceDimension,” and “orderCells” with the original default settings. Trajectories were visualized with “plot_cell_trajectory.” No quantitative metric of a trajectory “gap” or “discontinuity” was calculated. Pseudotime was therefore interpreted as an inferred transcriptional continuum rather than a direct measure of lineage progression or cellular differentiation.

Exploratory differential-expression and pathway-enrichment analysis

For exploratory nucleus-level characterization, differentially expressed genes (DEGs) within specified populations were obtained using Seurat “FindMarkers” with the Wilcoxon rank-sum test (“test.use = ‘wilcox’,” “min.pct = 0.1,” “logfc.threshold = 1.0”). Genes were classified as upregulated or downregulated according to the reported average log fold change. GO enrichment was evaluated using Metascape, and enrichment results were visualized with GOplot version 1.0.2 (The Comprehensive R Archive Network (CRAN), Vienna, Austria) [21]. KEGG enrichment was used for selected neuronal analyses.

Individual nuclei from the same donor are not independent biological replicates. Because donor-level pseudobulk re-analysis was not part of the original analytic record available for this revision, nucleus-level DEG P values were not interpreted as confirmatory disease-level inference. Instead, DEG and pathway results were treated as hypothesis-generating transcriptional associations. A donor-level pseudobulk analysis is required for definitive replication of disease-associated differential expression.

Transcriptional regulon analysis

Transcriptional regulatory network inference within astrocyte subsets was performed using SCENIC version 1.2.4 (Aerts Lab, VIB-KU Leuven Center for Brain & Disease Research, Leuven, Belgium) [22]. GENIE3 was used to infer transcription factor-target co-expression relationships, RcisTarget was used for motif-based pruning of candidate regulons, and AUCell was used to estimate regulon activity in individual nuclei. The regulon AUC matrix was binarized according to the SCENIC workflow and imported into Seurat for visualization. SCENIC results were interpreted as inferred regulatory activity rather than direct biochemical evidence of transcription-factor activation.

Image acquisition

DTI was acquired on a 3.0-T Discovery MR750 scanner (GE Healthcare, Milwaukee, Wisconsin). Parameters were as follows: slice thickness, 2 mm; no slice gap; 67 slices; 30 non-collinear diffusion-weighted gradient directions; maximum b value, 1,000 s/mm²; repetition time/echo time, 8,000/80.8 ms; flip angle, 90°; matrix, 128 × 128; native in-plane resolution, 2 × 2 mm²; and interpolation to 1 × 1 mm².

PET-CT was acquired on a Biograph mCT scanner (Siemens Healthineers, Erlangen, Germany). Participants fasted for at least six hours and received 3.7 MBq/kg 18F-FDG. Images were reconstructed using vendor-provided software with attenuation-weighted ordered-subsets expectation maximization. The reconstructed PET resolution was approximately isotropic, with a full width at half maximum of 4.4 mm at the center and 4.8 mm at 10 cm off-axis. Images were resliced to 2-mm sections.

DTI and PET-CT region-of-interest analysis

Diffusion imaging was processed using DSI Studio (Laboratory of Brain Anatomical MRI, Taipei Medical University, Taipei, Taiwan). DTI reconstruction was used to derive fractional anisotropy (FA) and mean diffusivity (MD) maps. Regions of interest (ROIs) were manually defined by experienced neurologists familiar with each patient's seizure semiology, EEG findings, and structural imaging. For each patient and modality, a 5-mm circular ROI was centered on the clinically defined epileptic lesion, and a corresponding ROI of the same size and shape was placed in the anatomically homologous contralateral region to provide an internal within-patient reference. Epileptic lesions were manually delineated using ITK-SNAP version 3.6.0 (Penn Image Computing and Science Laboratory, University of Pennsylvania, Philadelphia, Pennsylvania). PET signal intensity and DTI-derived FA and MD were measured in the lesion and contralateral reference regions.

The original analysis did not include atlas-based ROI placement or a formal inter-rater reliability analysis. These features are acknowledged as methodological limitations.

Statistical analysis

Statistical analyses were performed using IBM SPSS Statistics for Windows, Version 22 (Released 2013; IBM Corp., Armonk, New York) and R version 4.4.2 (R Foundation for Statistical Computing, Vienna, Austria). PET intensity, FA, and MD were compared between lesion and contralateral regions using paired t-tests, reflecting the within-patient paired design. Three imaging comparisons were prespecified; therefore, Bonferroni correction was applied, resulting in an adjusted significance threshold of P < 0.0167 (0.05/3). Formal effect-size estimates and confidence intervals were not part of the original imaging analysis record. Formal testing of the normality of paired differences was also not documented and is acknowledged as a limitation.

Cross-dataset interpretation strategy

Transcriptomic analyses were interpreted at the levels of cell-type composition, exploratory differential expression, pathway enrichment, pseudotime organization, and inferred transcription-factor regulon activity. Neuroimaging analyses were considered independent macroscopic phenotypes. Because the transcriptomic and imaging datasets were obtained from different cohorts, imaging findings were not used as direct molecular validation of the snRNA-seq findings. Cross-dataset interpretation was restricted to potentially concordant biological themes across different scales.

## Results

Study characteristics

Fifteen public PFC snRNA-seq samples were included: eight samples classified as epilepsy and seven controls (Table 1). The epilepsy group included five males and three females, with a mean age of 30.5 ± 11.3 years; the control group included four males and three females, with a mean age of 33.2 ± 13.2 years. All transcriptomic specimens were postmortem PFC samples. PMI ranged from 6 to 27 h in controls and from 18 to 39 h in epilepsy samples. Detailed epilepsy subtype and seizure-onset localization were not available.

**Table 1:** Public snRNA-Seq patient dataset PFC: prefrontal cortex; PMI: postmortem interval; RIN: RNA integrity number; SRA: Sequence Read Archive.

Sample name	Individual	Region	Age	Sex	PMI	RIN	Diagnosis	Capbatch	Seqbatch	Sequencing saturation	SRA accession
5787_BA9_Nova	5787	PFC	39	Male	16	7.9	Control	CB2	SB2	92.30%	SRX6034722
5609_BA9_Nova	5609	PFC	54	Female	6	8.5	Control	CB2	SB2	96.20%	SRX6034723
5546_BA9_Nova	5546	PFC	34	Female	27	8.3	Control	CB2	SB2	83.80%	SRX6034728
5981_BA9_Nova	5981	PFC	44	Female	19	7.8	Control	CB2	SB2	89.10%	SRX6034729
5958_BA9	5958	PFC	22	Male	24	7.6	Control	CB3	SB3	87.90%	SRX6033468
5577_BA9	5577	PFC	21	Male	19	8.6	Control	CB4	SB4	77.20%	SRX6033450
5893_BA9	5893	PFC	19	Male	11	8	Control	CB5	SB2	83.40%	SRX6034726
5839_BA9	5839	PFC	21	Female	18	8.6	Epilepsy	CB1	SB1	94.90%	SRX6034727
5873_BA9	5873	PFC	24	Male	39	8	Epilepsy	CB1	SB1	95.40%	SRX6034724
5881_BA9	5881	PFC	46	Female	36	8.1	Epilepsy	CB1	SB1	94.00%	SRX6034725
5876_BA9	5876	PFC	49	Male	26	8.6	Epilepsy	CB1	SB1	95.00%	SRX6034718
5929_BA9	5929	PFC	25	Male	25	7.8	Epilepsy	CB1	SB1	96.30%	SRX6034719
5850_BA9	5850	PFC	27	Female	30	7.7	Epilepsy	CB1	SB1	92.30%	SRX6034720
5892_BA9	5892	PFC	33	Male	25	8	Epilepsy	CB1	SB1	93.60%	SRX6034721
5607_BA9	5607	PFC	19	Male	29	8.1	Epilepsy	CB1	SB1	93.90%	SRX6034730

The independent imaging cohort included 16 patients with neocortical epilepsy (nine males and seven females; mean age 18.6 ± 7.4 years). Lesions were distributed equally between the left and right hemispheres and were identified using clinical MRI/PET findings in conjunction with EEG. The imaging cohort was younger than the transcriptomic cohort and did not overlap with it. 

snRNA-seq identifies multiple cell populations and exploratory differences in cellular representation

After quality control and integration, 16,132 nuclei were retained. Seventeen clusters were identified and visualized by t-SNE (Figure 1A). Major annotated populations included excitatory neurons, interneurons, astrocytes, oligodendrocytes, OPCs, endothelial cells, and microglia (Figure 1A,B). The distribution of nuclei by group is shown in Figure 1C-E.

**Figure 1:**
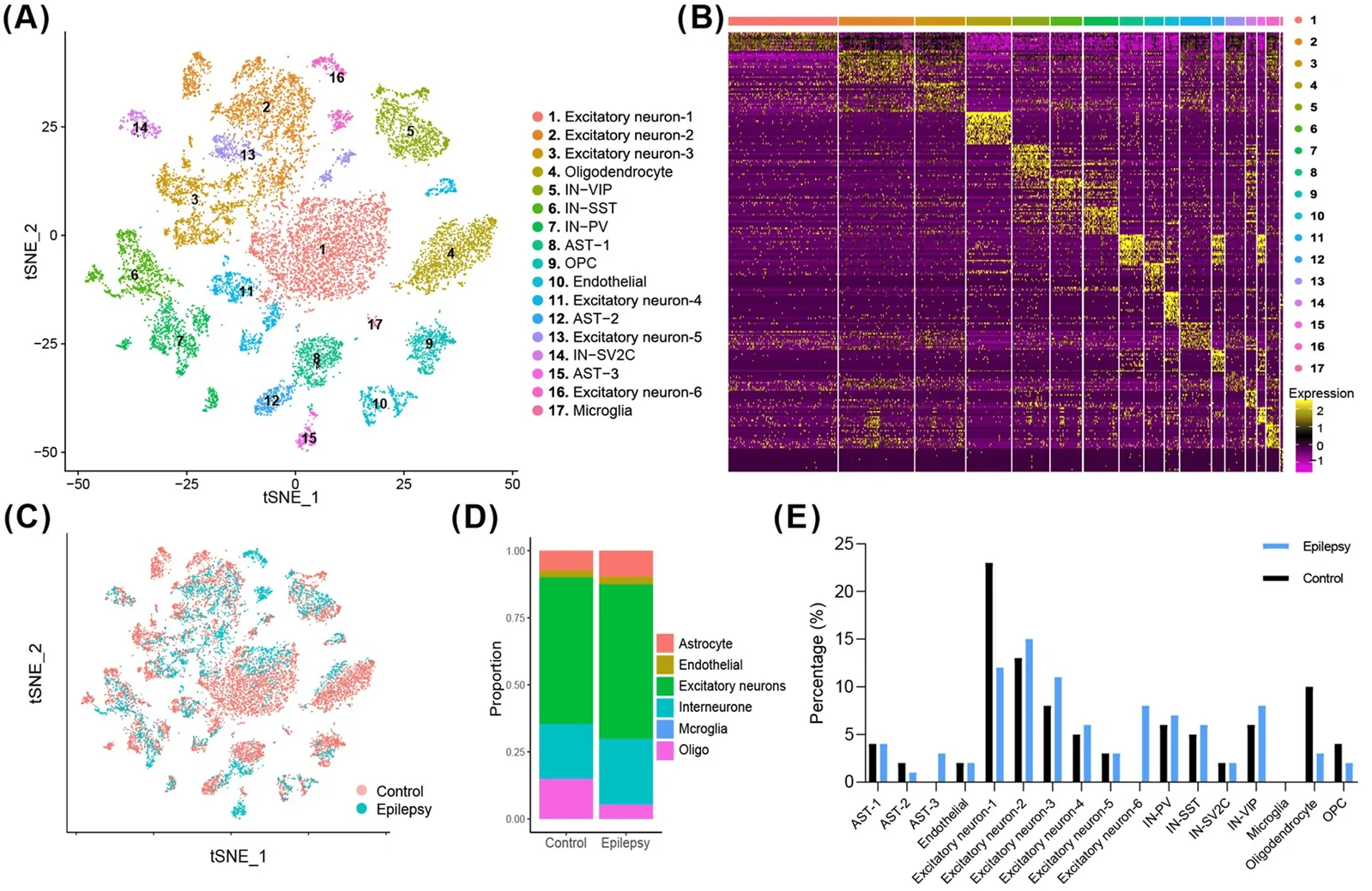
Clustering results of snRNA-seq data and the proportion of nuclei. (A) tSNE (t-distributed stochastic neighbor embedding) plot of 17 clusters obtained by clustering. (B) Heatmap of the 20 DEGs with the most varied expression in each cluster. (C, D) Proportion of the number of nuclei of major cell types in epilepsy and controls. (E) The proportion of nuclei in each cluster in epileptic and control groups. AST, astrocytes; IN-PV, parvalbumin interneurons; IN-SST, somatostatin interneurons; IN-SV2C, SV2C interneurons; IN-VIP, VIP interneurons.

In the original cell-composition comparison, oligodendrocyte-lineage nuclei represented 14.7% of control nuclei and 5.3% of epilepsy nuclei (exploratory p = 0.023). Additional descriptive differences were observed for AST-3, excitatory neuron clusters, oligodendrocytes, and OPCs. Because the total numbers of nuclei were unequal between groups and these comparisons were not based on donor-level compositional models, the proportions are presented as exploratory observations rather than definitive estimates of disease-associated abundance. Only 67 microglial nuclei were retained; therefore, microglia were not analyzed further.

L2/3 excitatory neurons show prominent transcriptional alterations and MAPK-associated enrichment

Excitatory neurons were reclustered and annotated using canonical cortical markers (Figure 2A,B). The resulting subgroups included NRGN-expressing neurons, L2/3 neurons, L4 neurons, L5/6 neurons, and L5/6 cortico-cortical projection neurons. Group distributions and exploratory nucleus-level DEG patterns are shown in Figure 2C,D. GO enrichment of the exploratory DEG set included terms related to synaptic signaling, ion transport, and energy metabolism (Figure 2E).

**Figure 2:**
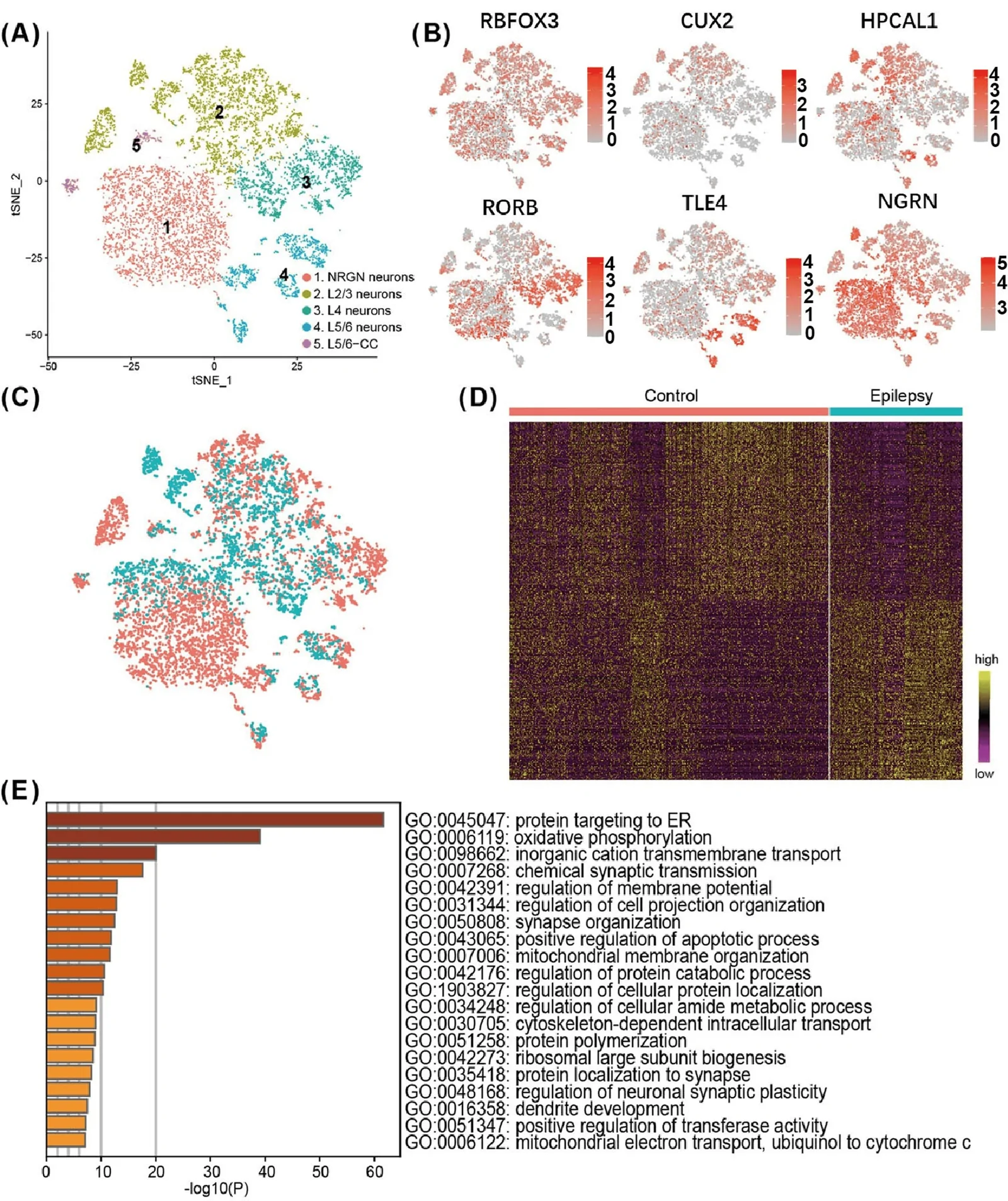
Results of regrouping of excitatory neurons and overall functional changes in epileptic samples. (A) tSNE (t-distributed stochastic neighbor embedding) plot of excitatory neuron subgroup clustering results. (B) The expression level map of subpopulation annotation signature genes. (C) tSNE plots were generated according to the grouping of samples by cell origin. (D) DEG heatmap was obtained by comparing the epilepsy group with control samples. (E) Display of BP entries obtained from GO analysis of the DEG heatmap. NRGN neurons, NRGN-expressing neurons; L2/3 neurons, layer 2/3 excitatory neurons; L4 neurons, layer 4 excitatory neurons; L5/6 neurons, layer 5/6 excitatory neurons; L5/6-CC, layer 5/6 cortico-cortical projection neurons.

L2/3 neurons represented 29.5% of excitatory neuronal nuclei in controls and 47.7% in epilepsy, whereas NRGN-expressing neurons represented 41.3% and 21.1%, respectively (Figure 3A). These descriptive patterns identify L2/3 neurons as one of the excitatory neuronal subtypes showing prominent differences in this dataset. Several activity-associated genes were expressed in L2/3 neurons (Figure 3B). In the exploratory KEGG analysis of upregulated L2/3 genes, the MAPK signaling pathway (hsa04010) was the top-ranked term, whereas ribosome-related pathways predominated among downregulated genes (Figure 3C,D). These findings indicate MAPK-associated transcriptional enrichment rather than direct evidence of pathway activation. 

**Figure 3:**
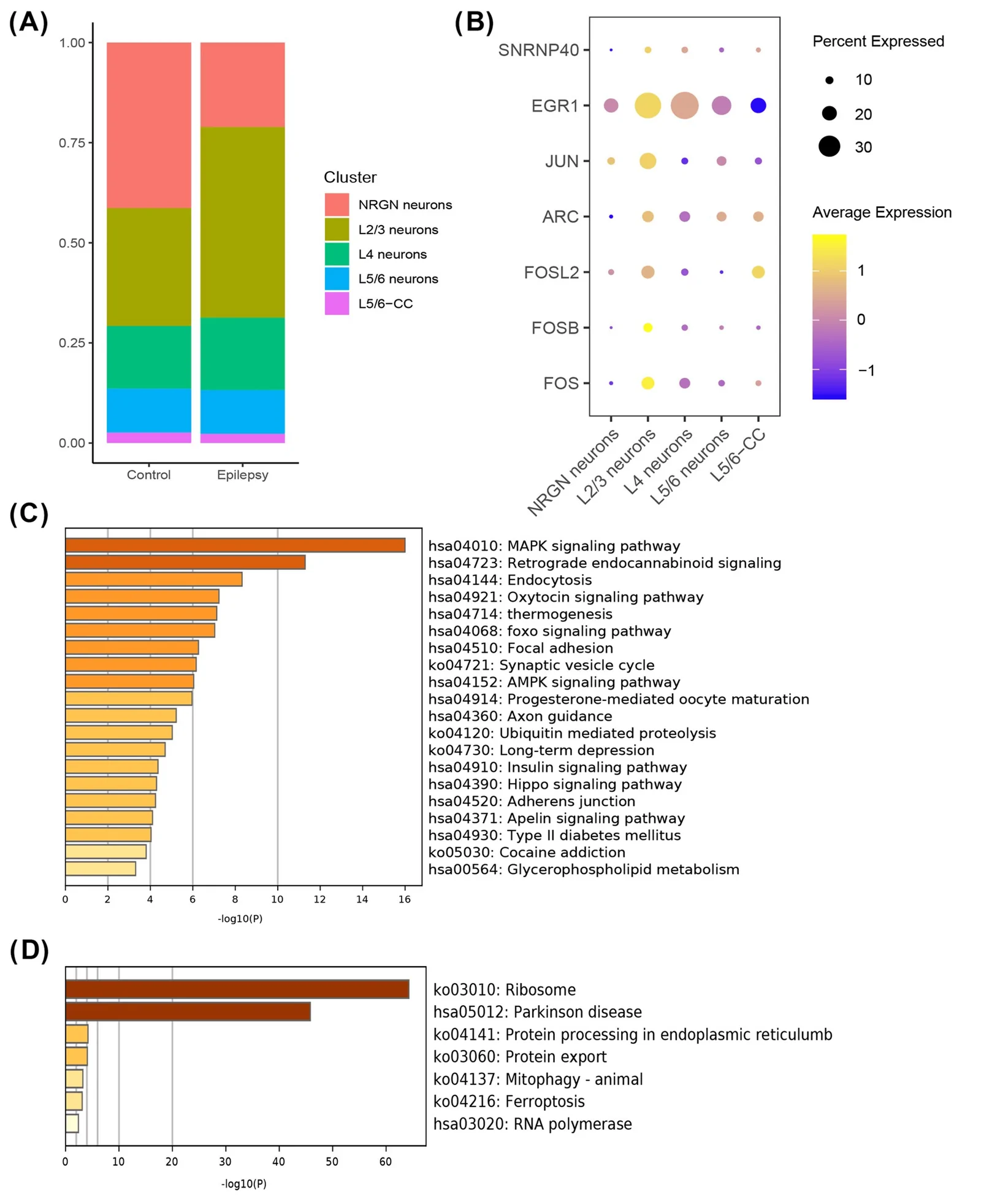
Identification of L2/3 excitatory neuronal subpopulations and analysis of the signaling pathways. (A) Proportion of each neuronal cluster in the epilepsy and control groups. (B) Expression of neuronal excitability-related genes in each cluster. (C) The upregulated DEG of cluster L2/3 neurons for KEGG pathway enrichment analysis. (D) The downregulated DEGs of cluster L2/3 neurons for KEGG pathway enrichment analysis.

Astrocytes exhibit STAT3-associated reactive transcriptional states

Astrocytes were reclustered into fibrous astrocyte (AST-FB) and protoplasmic astrocyte (AST-PP) populations, with additional epilepsy-enriched transcriptional clusters (Figure 4A-C). Exploratory DEG and GO analyses identified changes in genes and pathways related to cellular stress, metabolism, and growth (Figure 4D,E). Genes associated with reactive astrocyte states, including HSPA1A, HSPA1B, HSPB1, EMP1, VEGFA, CCL2, GFAP, and STAT3, showed heterogeneous expression across astrocyte subsets.

**Figure 4:**
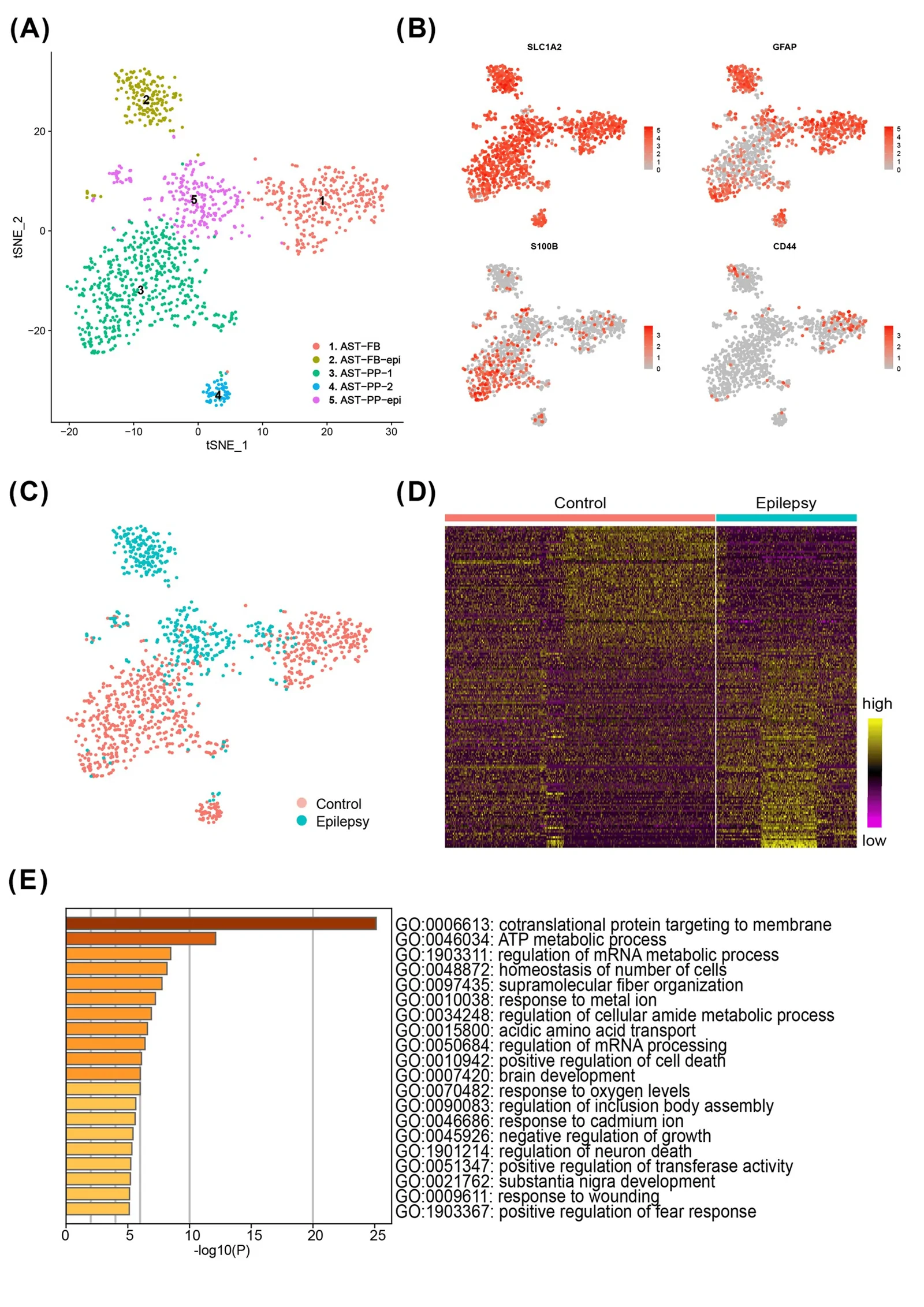
Results of regrouping of astrocyte and overall functional changes in epileptic samples. (A) tSNE (t-distributed stochastic neighbor embedding) plot of clustering results of astrocyte subpopulations. (B) The expression level map of characteristic genes of cell subpopulations. (C) tSNE plots were generated according to the grouping of samples by cell origin. (D) DEG heatmap was obtained by comparing the epilepsy group with control samples. (E) Display of BP entries obtained from GO analysis of the DEG heatmap. AST-FB, fibrous astrocytes; AST-PP, protoplasmic astrocytes; AST-FB-epi, epileptic fibrous astrocytes; AST-PP-epi, epileptic protoplasmic astrocytes.

SCENIC analysis identified an inferred STAT3 regulon pattern concentrated in AST-FB and epilepsy-enriched AST-FB nuclei (Figure 5). The expression pattern overlapped in part with previously described "A2-like" reactive signatures, but we do not interpret this as a discrete protective A2 phenotype because contemporary consensus recognizes multiple context-dependent reactive astrocyte states rather than a simple A1/A2 dichotomy. The present data therefore support a STAT3-associated reactive transcriptional state that requires functional validation.

**Figure 5:**
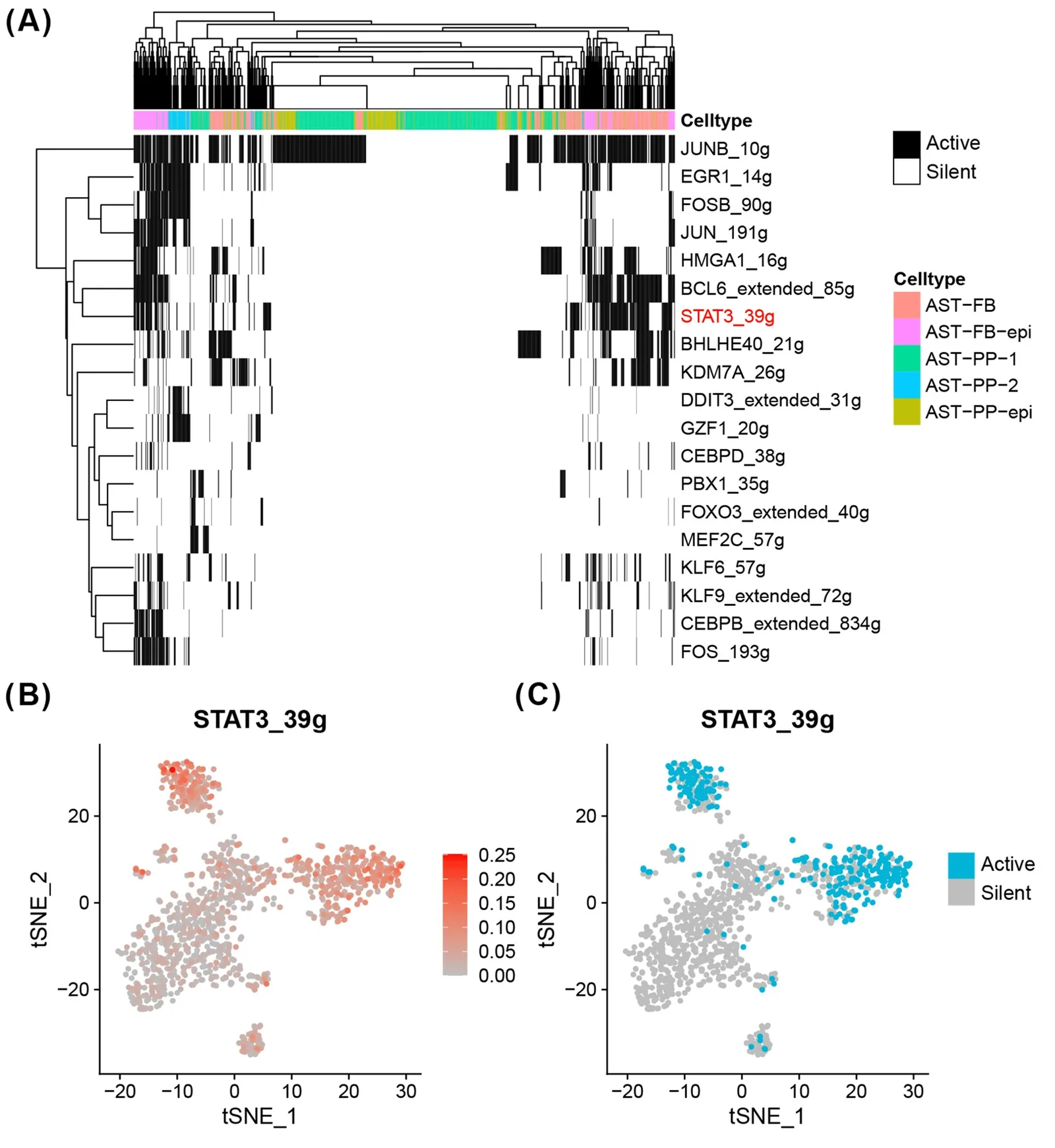
Results of astrocyte transcriptional regulatory network analysis. (A) The heatmap display of the results obtained by SCENIC analysis after binary conversion. (B) The expression map of the regulatory network was dominated by the transcription factor STAT3. (C) The expression of the transcription factor STAT3-dominated regulatory network after binary transformation.

Oligodendrocyte-lineage populations show altered transcriptional states

Oligodendrocyte-lineage nuclei were reclustered into oligodendrocyte and OPC-associated subpopulations (Figure 6A-C). Exploratory DEG and GO analyses identified transcriptional differences involving myelin-associated processes, cellular metabolism, membrane transport, and regenerative programs (Figure 6D,E). Gene-positive proportions differed for several oligodendrocyte- and OPC-associated genes, including MYRF, SEMA7A, CLCN3, NTN1, CSPG4, SEMA4D, TCF4, PDGFRA, NTRK2, NCAM1, and NCAM2 (Figure 7C-E). These gene-expression differences indicate altered oligodendrocyte-lineage transcriptional states but do not, by themselves, establish reduced proliferation, remyelination, or differentiation capacity.

**Figure 6:**
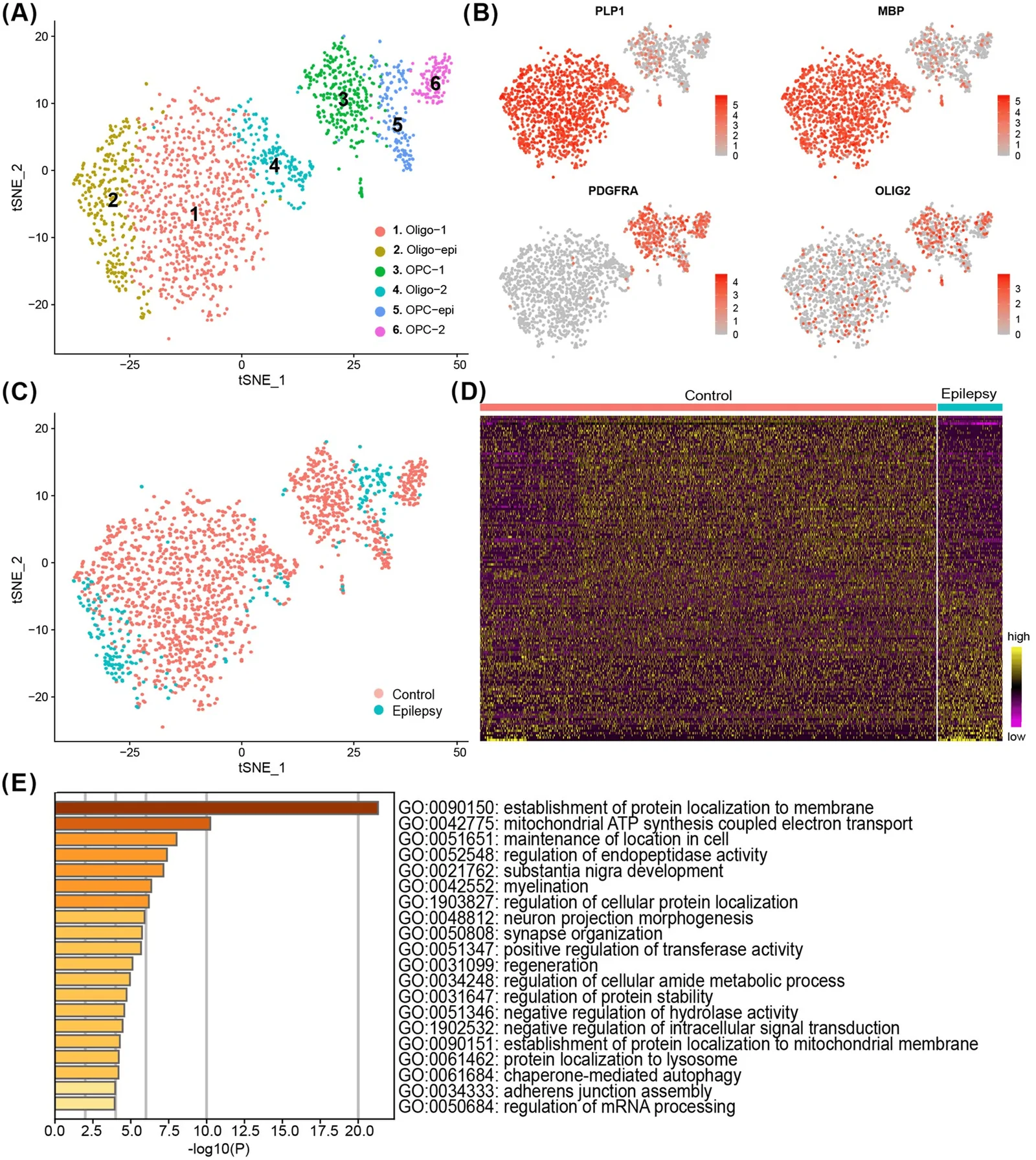
Oligodendrocyte lineage regrouping results and overall functional changes in epilepsy samples. (A) tSNE (t-distributed stochastic neighbor embedding) plot of clustering results of oligodendrocyte lineage subsets. (B) The expression level map of characteristic genes of cell subpopulations. (C) The t-SNE plot of tSNE according to cell-derived sample type. (D) DEG heatmap was obtained by comparing the epilepsy group with control samples. (E) Display of BP entries obtained from GO analysis of the DEG heatmap. DEG: differentially expressed gene; BP: biological process; GO: Gene Ontology; Oligo: oligodendrocytes; Oligo-epi: epileptic oligodendrocytes; OPC: oligodendrocyte precursor cell; OPC-epi: epileptic oligodendrocyte precursor cells.

**Figure 7:**
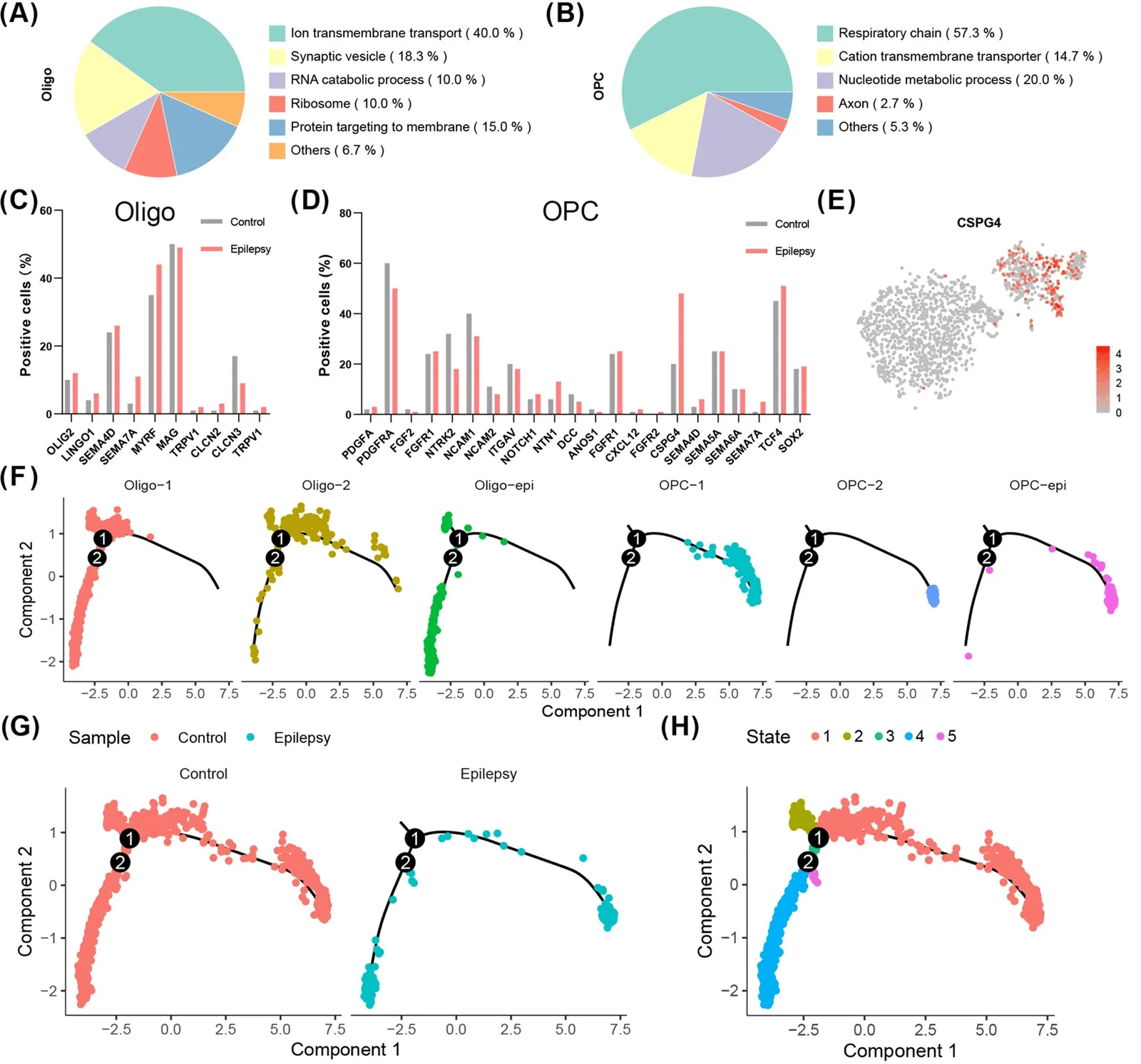
Functional changes in the oligodendrocyte lineage. (A, B) Gene Ontology (GO) functional enrichment analysis results. Terms with Z-score-transformed values ≤ −2 are shown. (C) Percentage of positive cells expressing genes related to oligodendrocyte function. (D) Percentage of positive cells expressing genes related to oligodendrocyte precursor cell (OPC) function. (E) Expression of the OPC marker gene CSPG4. (F) Pseudotime trajectory analysis of oligodendrocyte-lineage nuclei classified by cluster identity. (G) Pseudotime trajectory analysis classified according to sample group. (H) Distribution of nuclei across different pseudotime states before and after trajectory branching.

Pseudotime analysis reveals altered oligodendrocyte-lineage transcriptional organization

To examine transcriptional relationships across oligodendrocyte-lineage populations, pseudotime analysis was performed using OPC and oligodendrocyte nuclei. The inferred trajectory spanned OPC-like and oligodendrocyte-like transcriptional states (Figure 7F,G). Compared with controls, epilepsy nuclei showed a different distribution along the inferred continuum, with altered representation of specific states (Figure 7H).

Because pseudotime is an inference from cross-sectional transcriptional similarity rather than direct lineage tracing, and because substantially fewer oligodendrocyte-lineage nuclei were available in the epilepsy group (approximately 240 vs. 1,700 controls), the observed distribution should be interpreted cautiously. No quantitative trajectory-gap metric was calculated, and the analysis does not provide definitive evidence of impaired OPC differentiation.

Independent imaging cohort demonstrates metabolic and white matter microstructural abnormalities

PET-CT and DTI data from the independent cohort of 16 patients with neocortical epilepsy were analyzed using lesion-versus-contralateral comparisons. PET metabolic signal was lower in the lesion than in the corresponding contralateral region (P = 0.007; Figure 8A,B). FA was also lower in the lesion region (P < 0.001; Figure 8C,D). MD did not differ significantly between lesion and contralateral regions (P = 0.101; Figure 8E,F). After Bonferroni correction for the three prespecified imaging comparisons (P < 0.0167), the PET and FA differences remained statistically significant, whereas the MD comparison remained nonsignificant.

**Figure 8:**
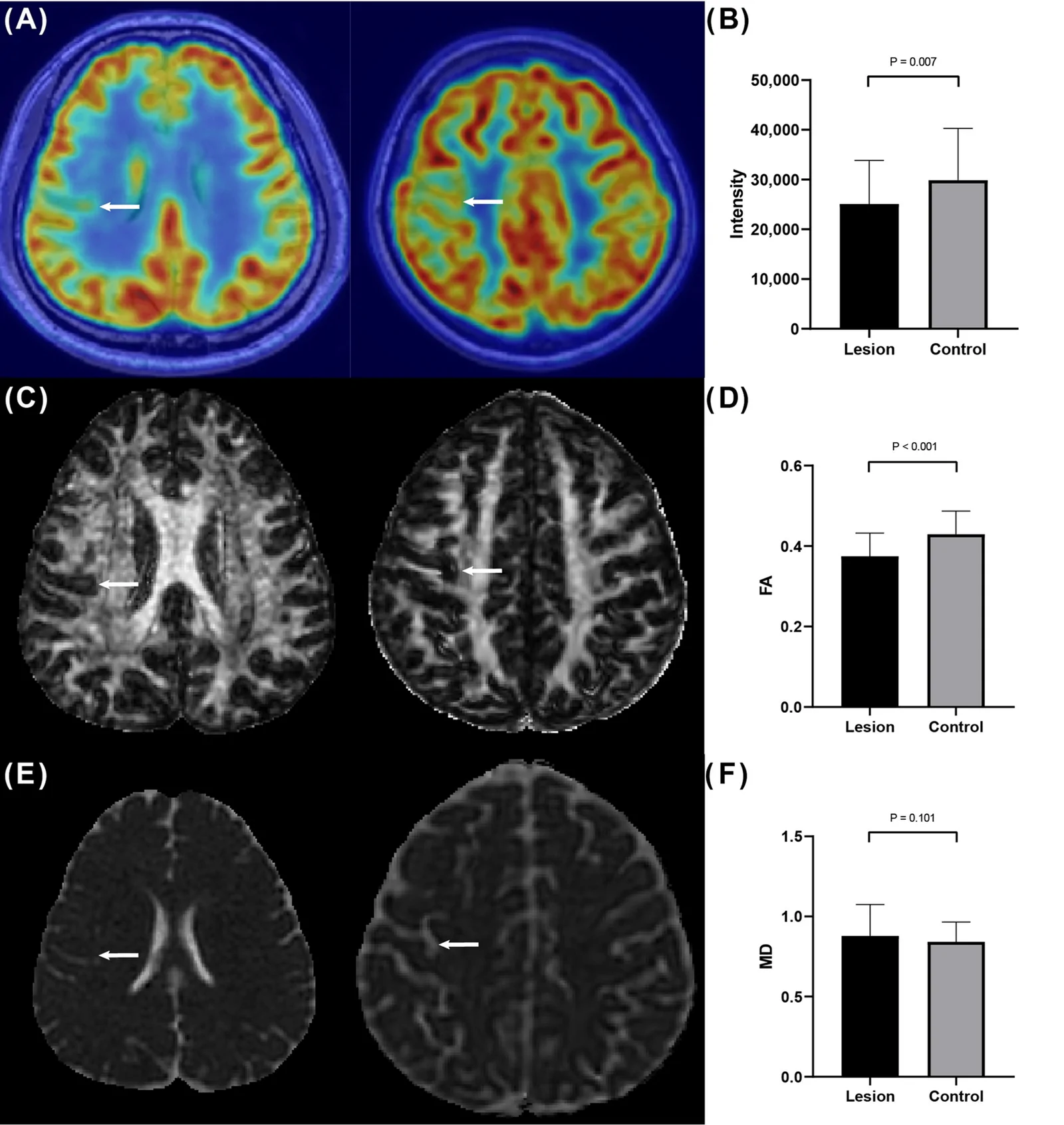
Comparison of PET-CT metabolic signal and DTI value in neocortical epilepsy patient. (A) PET-CT revealed hypometabolism in the patient’s lesion. (B) Quantification of the metabolic signal in the lesion area compared with the contralateral cortex. (C) The white matter around the patient’s lesion was marked on the FA value of DTI. (D) Quantification of the FA value of the lesion area compared with the subcortex on the contralateral cortex. (E) The white matter around the lesion was marked on the MD value of the DTI image. (F) Quantification of the MD value of the lesion compared with the subcortex on the contralateral cortex. PET-CT: positron emission tomography-computed tomography; FA: fractional anisotropy; DTI: diffusion tensor imaging; MD: mean diffusivity.

These findings demonstrate regional hypometabolism and altered white matter microstructural integrity in the clinical imaging cohort. They were not considered direct validation of the transcriptomic findings because the imaging and sequencing data were obtained from different individuals.

Cross-scale interpretation of transcriptomic and neuroimaging findings

The two study components identified abnormalities at different biological scales. The public PFC snRNA-seq analysis showed exploratory changes involving excitatory neuronal programs, STAT3-associated astrocytic states, and oligodendrocyte-lineage transcriptional organization. Independently, PET-CT and DTI demonstrated regional metabolic and white matter abnormalities in the clinical neocortical epilepsy cohort. These parallel observations are biologically compatible at a broad level but do not establish patient-level correspondence, direct molecular-imaging validation, or a causal mechanistic relationship.

## Discussion

Epilepsy is increasingly understood as a disorder of interacting neuronal, glial, and large-scale network processes. The present study examined these processes using two complementary but independent approaches: exploratory snRNA-seq analysis of a public postmortem PFC dataset and clinical PET-CT/DTI analysis in a separate neocortical epilepsy cohort. This framing is essential because the datasets differ in participants, age distribution, tissue context, and biological scale. Accordingly, our discussion focuses on the biological implications of each data stream and on broad cross-scale concordance rather than direct validation.

Our neuronal analysis identified prominent differences in L2/3 excitatory neurons. This observation is broadly consistent with Pfisterer et al., who reported epilepsy-associated neuronal subtype and gene-expression changes at single-cell resolution [13]. However, CUX2, a characteristic upper-layer excitatory neuronal marker emphasized in that work, was not significantly differentially expressed in our exploratory analysis. Differences between studies may reflect the cortical regions analyzed, epilepsy phenotypes, tissue acquisition, postmortem interval, sample composition, sequencing depth, or analytical strategy. The absence of CUX2 differential expression therefore does not establish either replication or contradiction of the previous study; instead, it highlights the potential region- and cohort-dependence of epilepsy-associated transcriptional signatures.

The exploratory L2/3 enrichment analysis identified MAPK-related signaling as the top-ranked KEGG pathway among upregulated genes. Human neocortical studies have previously linked epileptic cortical activity with persistent MAPK/ERK-CREB signaling, including spatially restricted changes in superficial cortical layers and L2/3 neurons [23,24]. These prior observations provide biological context for our enrichment result, but the present snRNA-seq analysis does not directly measure protein phosphorylation or biochemical pathway activation. We therefore interpret the result as a MAPK-associated transcriptional signature rather than evidence of pathway activation. Functional experiments and donor-level transcriptomic replication are required before inferring a mechanistic role.

Astrocytes showed heterogeneous epilepsy-associated transcriptional states, with an inferred STAT3 regulon concentrated in fibrous astrocyte-associated clusters. Astrocytes are known to influence seizure susceptibility through regulation of ions, neurotransmitters, metabolism, and inflammatory signaling [1,6,25]. Although some of the genes observed here overlap with signatures historically described as “A2-like,” contemporary consensus discourages a binary A1/A2 classification because reactive astrocytes adopt multiple context-dependent states that can simultaneously gain or lose different functions [25]. Consequently, our findings do not establish a protective astrocyte subtype. They instead identify a STAT3-associated reactive transcriptional state that warrants validation at the protein and functional levels. Recent work demonstrating disease-promoting reactive astrocyte states in epilepsy further illustrates the context dependence of astrocytic responses [26].

Oligodendrocyte-lineage cells also showed notable differences in representation and transcriptional organization. OPCs constitute a persistent proliferative population in the adult CNS and can generate oligodendrocytes throughout life [27,28]. Oligodendrocyte and myelin abnormalities have been described in focal cortical dysplasia and other focal epilepsy contexts [7]. In our dataset, oligodendrocyte-lineage nuclei were less represented and displayed changes in genes associated with myelin, membrane transport, metabolism, and OPC state. However, gene-positive percentages and pseudotime organization cannot directly demonstrate proliferation or differentiation capacity. The markedly smaller number of epilepsy oligodendrocyte-lineage nuclei may also alter trajectory density and state representation. Thus, the most defensible interpretation is altered oligodendrocyte-lineage transcriptional organization rather than a definitive differentiation block.

The apparent divergence between metabolism-related transcriptional enrichment and PET hypometabolism requires particular caution. Increased expression of metabolism-associated genes does not necessarily indicate increased tissue-level metabolic output. One possibility is that transcriptional changes represent compensatory responses to energetic stress or altered metabolic regulation rather than enhanced energy production. This explanation remains hypothetical and was not tested directly. In addition, snRNA-seq and PET assess different biological levels: snRNA-seq captures cell-type-specific transcriptional states, whereas 18F-FDG PET reflects integrated regional glucose utilization. Changes in cellular composition, neuron-glia metabolic coupling, or coordination between energy demand and supply could therefore produce different molecular and imaging signatures.

PMI adds another source of uncertainty to the metabolic interpretation. Both epilepsy and control snRNA-seq specimens were postmortem, but PMI distributions differed, with generally longer intervals in the epilepsy group. Postmortem interval and tissue ischemia can alter human transcriptomes [29]. Because PMI was not explicitly modeled in the original cell-level analysis, some metabolism- and stress-associated expression patterns may partly reflect postmortem effects. The transcriptomic and PET observations should therefore be regarded as complementary evidence of altered metabolic biology at different scales rather than mutually validating measurements.

The imaging results themselves are consistent with established observations in focal epilepsy. Interictal 18F-FDG PET commonly demonstrates hypometabolism in epileptogenic and propagation networks [16,30], and DTI studies have repeatedly documented reduced FA and, in many cohorts, increased MD in focal epilepsy [17,31]. In our imaging cohort, PET signal and FA differed significantly after correction for three prespecified comparisons, whereas MD did not. The nonsignificant MD result should not be interpreted as evidence of diffusivity abnormality. The use of the contralateral homologous region as a within-patient reference reduces some between-subject variability but does not remove limitations associated with manual ROI definition, seizure timing, or the absence of formal inter-rater reliability estimates.

Previous single-cell studies of epilepsy provide important context for these observations. Pfisterer et al. demonstrated substantial neuronal subtype-specific transcriptional alterations in epilepsy [13], whereas the present analysis extends the descriptive cellular landscape to astrocytic and oligodendrocyte-lineage states. At the same time, differences in brain region, clinical phenotype, tissue processing, and analytical framework mean that our findings should be considered complementary rather than a direct replication. Multi-cohort studies with harmonized clinical annotation and donor-level statistical inference will be required to identify reproducible disease-associated signatures.

Although the present analysis highlights candidate biological processes, these observations should not be interpreted as immediately actionable therapeutic strategies. MAPK-associated neuronal programs, STAT3-associated astrocytic states, and oligodendrocyte-lineage alterations are hypothesis-generating signals. Their causal relevance must be established using independent human cohorts, donor-aware statistical models, protein-level validation, and experimental manipulation. Systems-level studies of human epileptic tissue illustrate how transcriptional candidates can be followed by functional validation, but such validation was not performed here [32].

Future studies should therefore pursue three priorities. First, the transcriptomic findings should be re-evaluated in larger independent cohorts using donor-level pseudobulk or mixed-effects models, with detailed annotation of epilepsy subtype, seizure frequency, disease duration, medication exposure, seizure-onset region, and PMI. Second, matched molecular and imaging data from the same individuals would enable direct testing of relationships between cell-type-specific states and PET/DTI phenotypes. Third, experimental models and human tissue assays should test whether candidate MAPK-associated neuronal, STAT3-associated astrocytic, and oligodendrocyte-lineage programs contribute functionally to epileptic network dysfunction. If reproducible, such work may eventually support biologically informed patient stratification and more personalized approaches to epilepsy research.

Several limitations materially constrain interpretation of this study. First, the public snRNA-seq dataset lacked detailed clinical metadata regarding epilepsy subtype, seizure frequency, etiology, disease duration, treatment history, and whether the sampled PFC represented or was remote from the seizure-onset zone. The observed transcriptional patterns therefore cannot be attributed to a specific epilepsy syndrome or confirmed epileptogenic focus.

Second, the number of biological donors was small (eight epilepsy and seven control individuals), and the numbers of nuclei retained after quality control were markedly imbalanced. No group-level downsampling, bootstrap resampling, or donor-level pseudobulk analysis was performed in the original analysis. The use of nucleus-level Wilcoxon tests treats multiple nuclei from the same donor as independent observations despite their biological non-independence and can therefore underestimate between-donor variability. Accordingly, DEG P values, pathway enrichment, and cluster-composition comparisons are exploratory and require donor-level re-analysis before they can be considered confirmatory.

Third, pseudotime trajectories are inferred from cross-sectional transcriptional similarity and do not directly measure cellular lineage progression. No quantitative metric or permutation test for a trajectory gap was calculated. The lower number of epilepsy oligodendrocyte-lineage nuclei may influence trajectory density and the detection of specific states. Therefore, pseudotime differences cannot be interpreted as definitive evidence of impaired OPC differentiation.

Fourth, both transcriptomic groups were postmortem, but PMI differed between groups and was not included as a covariate in the original analysis. PMI-related RNA degradation and postmortem transcriptional changes may have influenced metabolism- and stress-associated signatures.

Fifth, the transcriptomic and imaging cohorts were independent and differed substantially in age and clinical composition. There was no patient-level correspondence between the public adult PFC dataset and the younger local imaging cohort. Direct molecular-imaging correlation or validation is therefore impossible, and apparent concordance may partly reflect cohort-specific or age-related factors.

Sixth, the imaging cohort was small. ROIs were manually delineated, formal inter-rater reliability was not quantified, normality testing of paired differences and formal effect-size estimates were not documented in the original analysis, and detailed timing of PET acquisition relative to the most recent seizure was not available in the manuscript record. Because seizure timing can influence interictal FDG-PET interpretation [30], these factors limit quantitative generalizability.

Finally, no independent transcriptomic cohort or wet-laboratory experiment was used to validate the MAPK-associated neuronal programs, STAT3-associated astrocytic states, or oligodendrocyte-lineage alterations. These findings should therefore be considered hypothesis-generating until reproduced using donor-level statistical frameworks and functional experimental approaches.

## Conclusions

In conclusion, this two-part exploratory study provides complementary perspectives on epilepsy-associated brain alterations. The public postmortem PFC snRNA-seq analysis identified cell-type-specific transcriptional patterns involving L2/3 excitatory neurons, STAT3-associated reactive astrocytic states, and oligodendrocyte-lineage populations, whereas an independent clinical imaging cohort demonstrated regional hypometabolism and reduced white matter FA. Because the two datasets were obtained from different populations, these observations do not constitute direct molecular-imaging validation. The transcriptomic results also require donor-level re-analysis to address pseudoreplication. Future work using larger clinically annotated cohorts, donor-aware statistical models, matched molecular-imaging measurements, and functional experiments will be necessary to determine which of these candidate cellular programs are reproducible, mechanistically relevant, and clinically informative.
